# Lightweight SCC Development in a Low-Carbon Cementitious System for Structural Applications

**DOI:** 10.3390/ma16124395

**Published:** 2023-06-14

**Authors:** Galal Fares, Ahmed K. El-Sayed, Abdulrahman M. Alhozaimy, Abdulaziz I. Al-Negheimish, Abdulrahman S. Albidah

**Affiliations:** 1 Center of Excellence for Concrete Research and Testing, Department of Civil Engineering, College of Engineering, King Saud University, P.O. Box 800, Riyadh 11421, Saudi Arabia; ahelsayed@ksu.edu.sa (A.K.E.-S.); alhozimy@ksu.edu.sa (A.M.A.); negaimsh@ksu.edu.sa (A.I.A.-N.); 2 Department of Civil Engineering, College of Engineering, King Saud University, P.O. Box 800, Riyadh 11421, Saudi Arabia; aalbidah@ksu.edu.sa

**Keywords:** lightweight aggregate, scoria rocks, mixing protocol, rheological properties, mechanical properties

## Abstract

The utilization of manufactured lightweight aggregates adds another dimension to the cost of the preparation of self-compacting concrete (SCC). The common practice of adding absorption water to the lightweight aggregates before concreting leads to inaccurate calculations of the water-to-cement ratio. Moreover, the absorption of water weakens this interfacial bond between aggregates and the cementitious matrix. A particular type of black volcanic rock with a vesicular texture known as scoria rocks (SR) is utilized. With an adapted sequence of additions, the occurrence of water absorption can be minimized to overcome the issue of calculating the true water content. In this study, the approach of preparing the cementitious paste first with adjusted rheology followed by the addition of fine and coarse SR aggregates enabled us to circumvent the need for adding absorption water to the aggregates. This step has improved the overall strength due to the enhanced bond between the aggregate and the cementitious matrix, rendering a lightweight SCC mix with a target compressive strength of 40 MPa at 28 days, which makes it appropriate for structural applications. Different mixes were prepared and optimized for the best cementitious system that achieved the goal of this study. The optimized quaternary cementitious system included silica fume, class F fly ash, and limestone dust as essential ingredients for low-carbon footprint concrete. The rheological properties and parameters of the optimized mix were tested, evaluated, and compared to a control mix prepared using normal-weight aggregates. The results showed that the optimized quaternary mix satisfied both fresh and hardened properties. Slump flow, T50, J-ring flow, and average V-funnel flow time were in the ranges of 790–800 mm, 3.78–5.67 s, 750–780 mm, and 9.17 s, respectively. Moreover, the equilibrium density was in the range of 1770–1800 kg/m^3^. After 28 days an average compressive strength of 42.7 MPa, a corresponding flexural load of over 2000 N, and a modulus of rupture of 6.2 MPa were obtained. The conclusion is then drawn that altering the sequence of mixing ingredients becomes a mandatory process with scoria aggregates to obtain high-quality lightweight concrete for structural applications. This process leads to a significant improvement in the precise control of the fresh and hardened properties, which was unachievable with the normal practice used with lightweight concrete.

## 1. Introduction

Lightweight concrete (LC) is distinguished from conventional concrete by its low density, which ranges from 1440 to 1840 kg/m^3^, while conventional concrete with its normal weight is identifiable by its regular density above 2000 kg/m^3^ [1,2]. Lightweight aggregates are generally divided into manufactured and natural types. Lightweight aggregates can be obtained from local natural materials such as scoria and pumice stones or manufactured as lightweight expanded clay aggregate (LECA) from expanded clay shale with added cost [3,4,5,6]. Manufactured lightweight aggregates increase the cost of concrete production and, in some cases, create environmental overburden [7]. The mechanical properties of concrete formed with lightweight aggregates are affected by the type and water content of the aggregates [8]. The main disadvantages of lightweight aggregates in concrete are their high cost, poor mechanical properties, and general durability concerns. However, LC has its own advantages and is preferred in the construction industry. Different types of lightweight concrete are produced, such as lightweight aggregate concrete (LWAC), aerated concrete (AC), and no-fines concrete (NFC). The main idea behind these types is the creation of a large system of void content in the concrete skeleton [7,9]. The oven-dry density of lightweight concrete is reported to range from 300 to less than 2000 kg/m^3^, and its cube strength is up to 60 MPa [10,11]. Similarly, lightweight concrete is classified into three types based on its strength: low, moderate, and structural strength concrete, with the latter ranging from 17 to 63 MPa [11,12]. There are numerous advantages to using LC in construction [13]. The reduction of formwork and the resulting dead loads, as well as the improved thermal resistance, account for the majority of the advantages of producing lightweight concrete [14,15]. In addition to the mentioned benefits, there are many other benefits; however, the difficulties in controlling the water content, casting in situ, and finishing represent the main downsides. Many researchers have proposed the production of self-compacting lightweight aggregate concrete (SCLWAC) to overcome these issues [16,17,18,19,20]. Large deposits of scoria rocks are spread along the Arabian Shield. Various studies have shown that these deposits have the potential to be used as lightweight aggregates and natural pozzolanic materials [21,22,23,24]. To reduce the carbon footprint caused by the production of cement, it becomes morally required and necessary to use more cementitious ingredients in large quantities of concrete without compromising its properties. Due to the enormous demand and lack of effective substitutes, the current construction boom’s heavy usage of silica fume, fly ash is insufficient to compensate for the uncontrolled manufacture of cement; namely, the extremely large quantities cannot be covered by them. In this study, it was possible to develop a quaternary cementitious system utilizing cement, silica fume, fly ash, limestone dust (LSD) from aggregate quarries and the fine powder found in scoria rocks. The current study demonstrates an unconventional method for employing readily available materials such as scoria rocks reinforced with an optimized quaternary cementitious system to lessen cement’s environmental impact and boost the effectiveness of specially formulated composites to formulate lightweight concrete with a low carbon footprint. Moreover, the proposed procedure allows the accurate estimation of mixing water. Accordingly, the utilization of abundantly available and cost-effective scoria rocks as a natural alternative to manufactured aggregates is feasible to investigate. In this study, scoria rocks (SR) were collected from the western part of the Arabian Peninsula, where they are extensively found, covering an area of about 180,000 km^2^ [25]. Scoria rocks were used in the production of ternary lightweight concrete containing fly ash and silica fume, where the ultimate strength of about 33 MPa was obtained after 91 days [26]. Scoria rocks were also used as the primary aggregates in that study. In another study, the utilization of different types of lightweight aggregates, such as scoria, perlite, and Leca was conducted in the production of SCC mixes [3]. In that study, lightweight aggregates replaced normal aggregates with up to 60% of their weight. Until 28 days had transpired, the maximum compressive strength in all mixes was less than 35 MPa. In the current study, the testing program is divided into various parts that discuss different parameters and issues that hinder the delivery of the final mix. The influence of the water content, if the scoria aggregates have varied moisture contents, on the concrete properties is explored in this study. The major objectives of this study are to produce a final mix with a precise water-to-binder ratio, SCC flowability, and target hardened properties by examining the parameters impacting the unit weight and the effectiveness of the desired flowability, including the impact of superplasticizer. The main aim of the current study is, accordingly, to formulate an optimized lightweight SCC mix for structural applications with a dry unit weight below 1700 kg/m^3^ using 100% fine and coarse scoria rock aggregates.

## 2. Materials, Characterization and Testing Scheme

**Figure 1:**
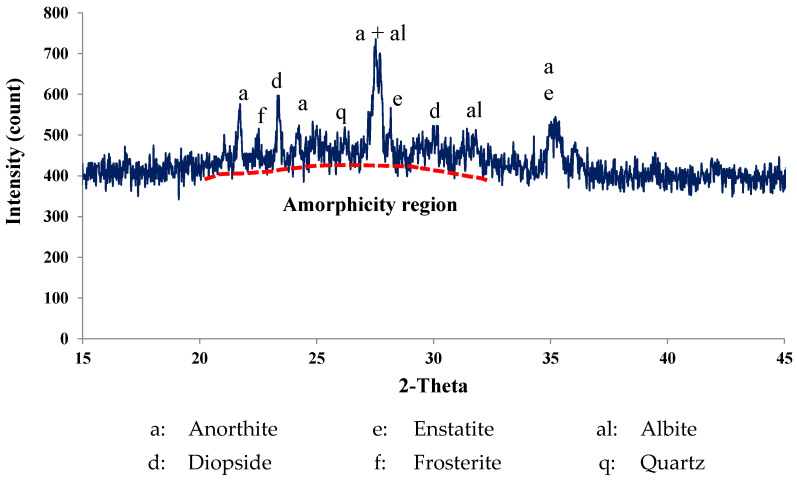
XRD analysis of the ground SR sample.

**Figure 2:**
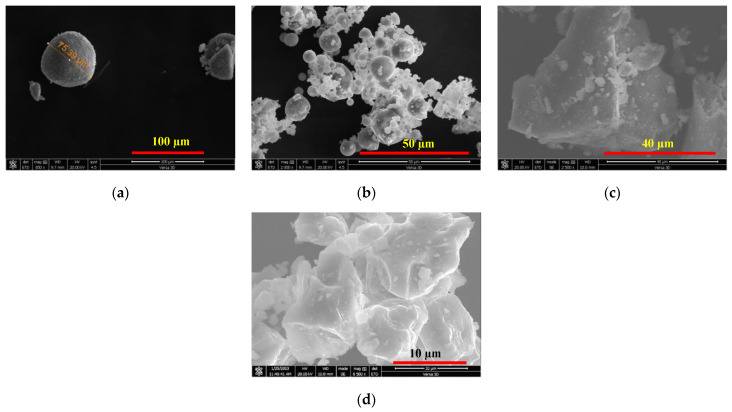
SEM photomicrograph of (**a**) SF, (**b**) FA, (**c**) ground SR and (**d**) LSD powders.

**Figure 3:**
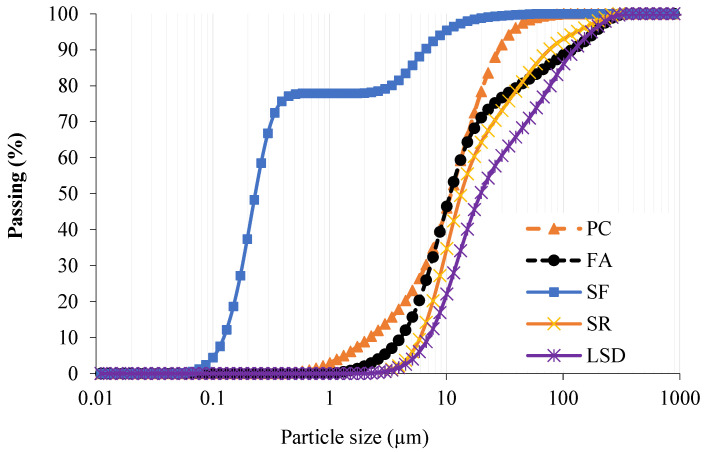
Particle size distribution of PC and fine powders.

**Figure 4:**
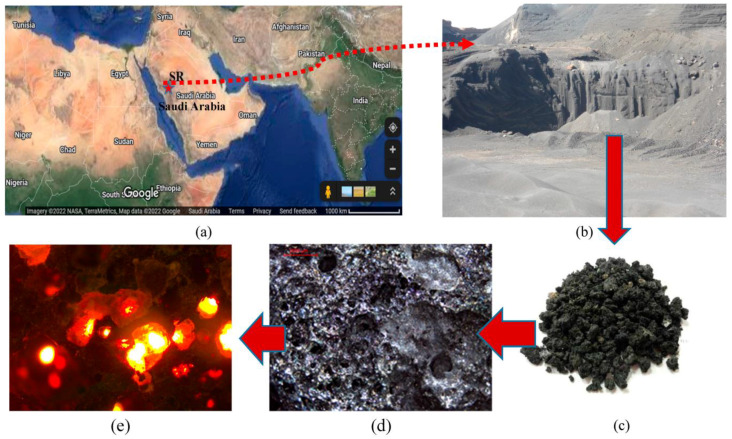
Illustration of the collection site; (**a**) location of site on Google maps, (**b**) actual volcanic lava field, (**c**) visually represented grains and (**d**) microscopically illustrated showing (**e**) scattered light through the vesicles that caused lightweight.

Different types of cementitious materials were used as the main binder for the preparation of SCC mixes. The cementitious materials and fine powders were ordinary Portland cement (PC), silica fume (SF), class F fly ash, and limestone dust obtained from local aggregate crushers (Riyadh crushers, Riyadh, Saudi Arabia). Limestone dust (LSD) powder was procured from stone crushers in the Rumah area, northeast of Riyadh. PC meets the ASTM C 150 specification [27], whereas SF and FA meet the ASTM C1240 and C618 specifications, respectively [28,29]. Scoria rocks (SR) were collected from the Arabian Peninsula’s western coast. The X-ray fluorescence (XRF), model Cemoxi, and the X-ray diffraction (XRD), model Empyrean, analyses (PANalytical, Almelo, Netherlands) of the same powder provided their chemical and mineralogical compositions, as presented in Table 1 and Figure 1, respectively. The physical properties of the fine powder, including the median size (D50) and specific gravity, are also shown in Table 1. The XRD analysis reveals that SR samples contain pyroxene, feldspars, and plagioclase minerals. The scanning electron microscopy (SEM) (Versa-3D dual-beam field emission scanning electron microscope from FEI, North Brabant, Netherlands) analysis provided insight into the microstructure of the powder, as demonstrated in Figure 2. The laser particle-size distribution of fine powders was calculated using the LA-950 analyzer from Horiba (Horiba, Paris, France) and is presented in Figure 3. The procurement latitude coordinates from the Global Positioning System and collection site information for scoria rocks are provided in Table 2 and Figure 4, respectively.

**Figure 5:**
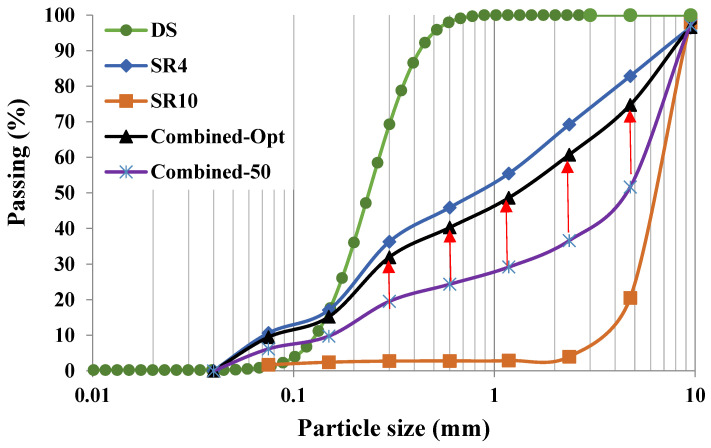
Sieve analysis of the individual aggregate and combined forms.

**Figure 6:**
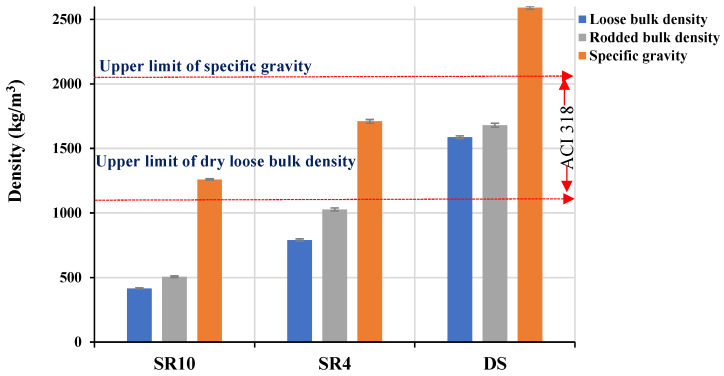
Physical properties of fine and coarse aggregates, as per ACI 318 range [30].

In this study, different SRs of nominal sizes 4 and 10 mm were used to obtain low-density concrete. Moreover, the use of dune sand (DS) as fine aggregates was investigated in this study in the optimization tests to evaluate its effect on the density of concrete. The sieve analysis and physical properties of SR and DS aggregates are given in Figure 5 and Figure 6, respectively. Figure 5 shows that DS is finer than SR4 and SR10, which may affect the compactness of concrete and consequently increase its density. SR4 had a better grain distribution. The optimization of aggregate gradation was critical, as it affects both the fresh properties and the hardened properties, as well as the unit weight. For example, an equivalent combination of SR4 and SR10 (50% SR4 + 50% SR10) provided a coarser gradation, which is identified as Combined-50, as shown in Figure 5. However, the optimum combination (Combined-Opt) could be obtained by mixing 13% SR10 with 87% SR4, as demonstrated in Figure 5. The loose and rodded bulk densities as well as the particle densities of each type of aggregate are shown in Figure 6. The fine content below 75 µm was about 9% in SR4 and 1.7% in SR10. The physical properties of both the fine and coarse aggregates, such as specific gravity and loose and rodded bulk densities compared with ACI 318 limits [30], are shown in Figure 6. The water absorptions for SR4, SR10, and DS were 14, 22, and 0.38%, respectively.

**Figure 7:**
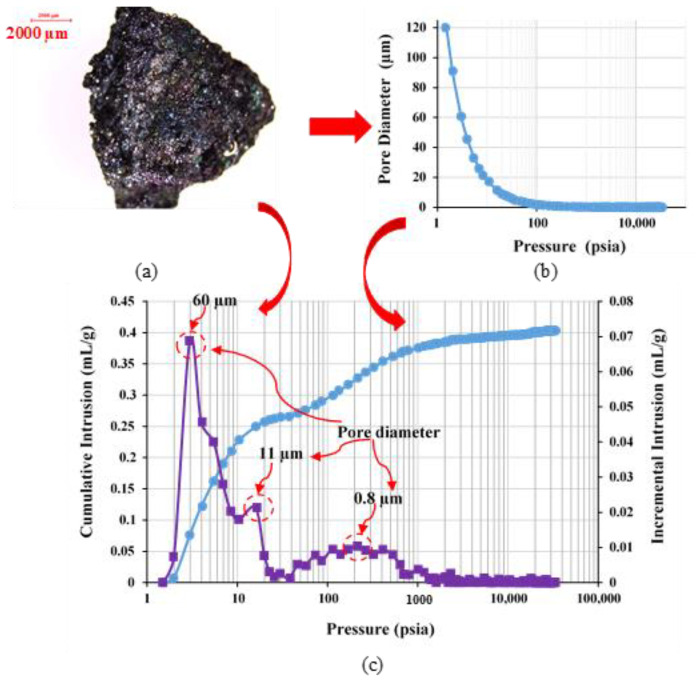
MIP analysis of (**a**) SR grains showing (**b**) the pore diameter distribution and (**c**) the cumulative intrusion as a function of applied pressure. Both (**b**,**c**) are correlated with same applied pressure.

**Figure 8:**
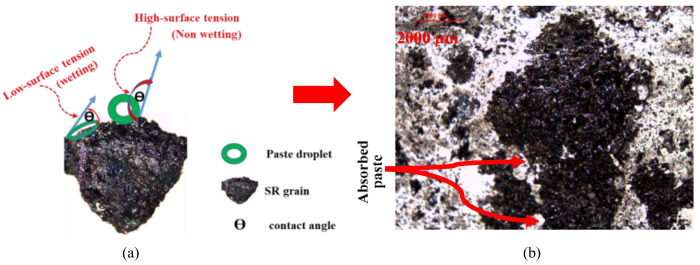
The wet (**a**) SR coarse aggregate has the ability to (**b**) absorb the cementitious matrix and affect workability.

Mercury intrusion porosimetry (MIP) analysis of SR grains is shown in Figure 7. According to the MIP analysis, the critical pore size is 60 μm, which makes absorption of the cementitious paste with a low water-to-cement ratio difficult due to the elevated surface tension accompanied by elevated plastic viscosity, as illustrated in Figure 8. The very fine porous system in fine and coarse SR aggregates proved beneficial to avoid the addition of absorption water to the aggregate, and accordingly, the exact water content could be estimated. The utilization of polycarboxylateether-based (PCE) superplasticizer was the main chemical admixture in this study. PCE dosage is expressed as a dry extract in terms of a percentage of binder content.

**Figure 9:**
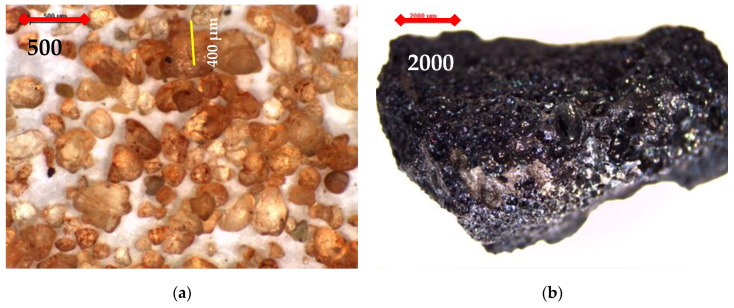
Photomicrographs of (**a**) dune sand (35×) and a scoria rock grain (8×) (**b**).

For trial concrete mixes, a blend of SR4 and natural dune sand (DS) complying with ASTM C33 [27,31] was also used as fine aggregate for unit weight, as well as different blends of SR4 and SR10, as demonstrated in Figure 9a,b. The microscopic investigation of DS has confirmed the sphericity of its particles compared to the sponge-like structure of scoria grains, as shown in Figure 9a,b, respectively.

### 2.1. Testing Scheme

The testing program is divided into various parts that discuss different parameters and issues that hinder the delivery of the final mix. The effect of the water content, if the scoria aggregates have different moisture contents, on the concrete properties is covered in this study. The parameters affecting the unit weight and the quality of the desired flowability, including the influence of superplasticizer, are the main goals of the study to reach the final mix with an accurate water-to-binder ratio, SCC flowability, and target hardened properties. The final mix obtained is under investigation for structural applications in reinforced concrete beams.

#### 2.1.1. Effect of Aggregate Water Content

The effect of the water content of SR aggregates is very critical in defining the water-to-binder ratio accurately. In this study, different water contents of 0, 25, 65, and 100% of SR aggregates were investigated for the flowability of concrete mix using a modified mini-slump test. The optimum content was defined according to this test.

##### Unit Weight Approach

The unit weight was the primary criterion for considering the concrete mix for further investigation in this section of the study. The use of dune sand along with SR4 and SR10 was assessed first, followed by 100% utilization of various blends of SR4 and SR10.

#### 2.1.2. Effect of Coarse-to-Total Aggregate Ratio on Flowability

The optimal combination of SR4 and SR10 was investigated in various combinations with different SR10 weight ratios to total aggregates (SR10/(SR4+SR10)). These ratios varied from 0.125 to 0.2.

#### 2.1.3. Adjusting Superplasticizer Dosage

The optimization of superplasticizer dosage is critical as it affects the rheological properties as well as the stability of the concrete mix. The effect of different dosages on flowability and surface stability was investigated to determine the optimum superplasticizer dosage.

### 2.2. Final Mix

After optimizing the parameters defining the mix composition of the final mix, the rheological properties of the cementitious paste composing the concrete mix and final concrete mix were investigated. The temperature profiles of the cementitious and concrete phases were determined in this part of the study.

#### 2.2.1. Paste Mixes

**Figure 10:**
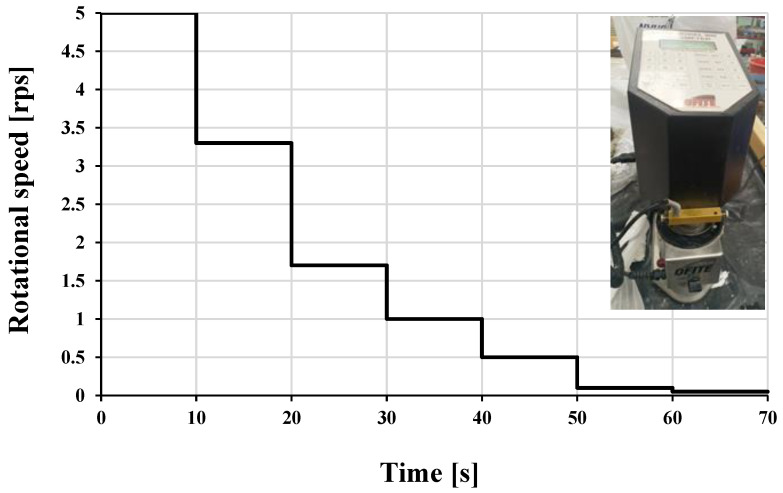
Measuring down steps used for the rheological properties of cementitious paste composing LWSCC mix.

Different paste mixes composing the concrete mix shown in Table 3 were investigated for rheological properties using an Ofite Model 900 viscometer with the measurement steps shown in Figure 10. It is a type of rotational viscometer with the geometry of a true Couette coaxial cylinder with a shear rate range from 0.01–1700 s^−1^ and speed accuracy of 0.001 RPM. The temperature profile of the same paste mix was determined.

#### 2.2.2. Concrete Mixes

The low-carbon optimum mix composition of the quaternary cementitious matrix contained 55% cement, 18.5% FA, 7% SF, and 19.6% LSD, while the aggregate volume of the total volume was about 45%. The fine SR aggregate made up about 87.2% of the total aggregate weight. The optimum dosage of superplasticizer is defined in this part of the study. Different concrete mixes with the mix composition shown in Table 3 were prepared and tested for unit weight using DS, SR4, and SR10.

Based on the physical properties of aggregates and the adopted concept of mixing, the specific sequence of adding materials becomes as follows:

- Homogenize the cementitious binder (cement followed by fly ash, silica fume, and limestone dust) for 5 min of dry mixing;
- Add water with chemical admixture and mix for 6 min;
- Add fine aggregates and mix for another 4 min until flowable, followed by a 1-min pause to add coarse aggregate and mix for another 6 min or more, will guarantee the desired apparent flowability characteristic of the SCC mix.

**Figure 11:**
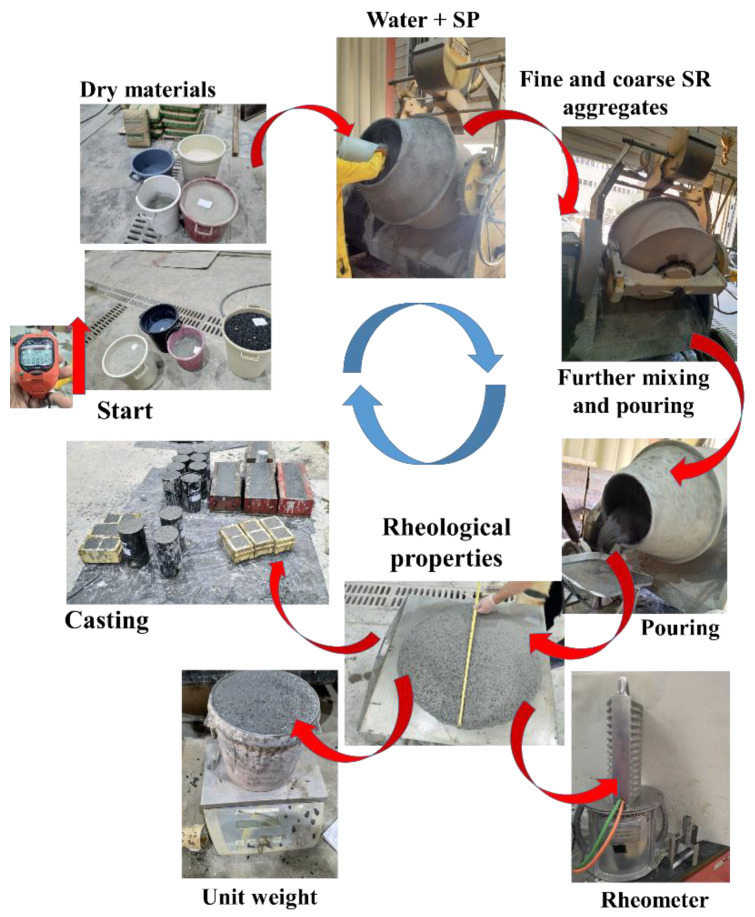
Graphical representation of mixing protocol arrows to be followed from start to casting.

The fresh mix was then tested for unit weight, rheological properties, and the temperature profile, and then the mix was cast into a 2-inch cube and two sizes of molds (40 × 40 × 160 mm and 150 × 150 × 600 mm) for compressive and flexural properties using the test setups. The whole process is visualized in Figure 11.

**Figure 12:**
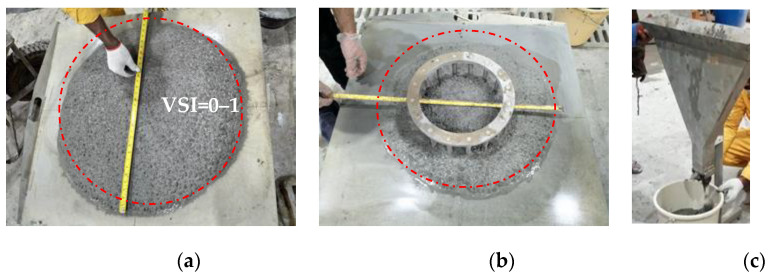
Measuring (**a**) slump flow and assessing SCC stability using ASTM C1611 [32] (**a**) mix slump flow with acceptable visual stability indices ((VSI = 0–1)), (**b**) J-ring flow test and (**c**) V-funnel flow time test.

Typical test setups for fresh properties are shown in Figure 12. Slump flow, slump flow time (T50) [32], visual stability index (VSI), J-ring flow, and V-funnel flow test were the rheological parameters investigated in this study, as demonstrated in Figure 12a–c, respectively. The formulated LWSCC mix composition has been tested in different batches with VSI ranges from 0 to 1, as an indication of the high degree of stability of the mixes.

**Figure 13:**
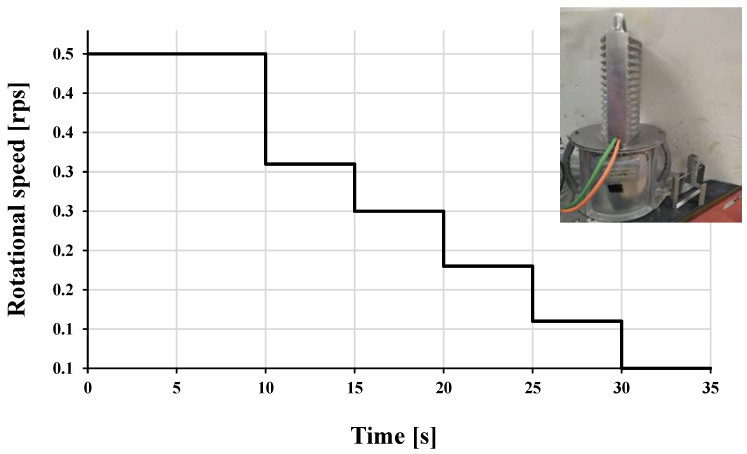
Measuring down steps sequence used for the rheological properties of LWSCC mix using the ConTec Rheometer-4SCC with rotary vane of.

A sub-version of the Rheomixer, the ConTec Rheometer-4SCC with Rotary Vane, was used in this study to investigate the LWSCC final mix with the measuring sequence shown in Figure 13. It has a concrete capacity of 7 L to perform the necessary test. The filling time, measuring, and data processing necessitate less than 2 min.

**Figure 14:**
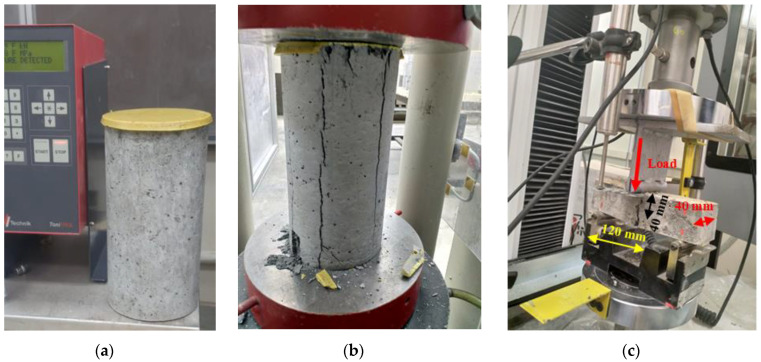
Experimental setup for testing compressive (**a**) before and (**b**) after testing and (**c**) flexural properties of prism specimens.

The test setups for the compressive and flexural properties are shown in Figure 14. A 3000 kN universal testing machine with a compression loading rate of 0.2 MPa/s as per ASTM C109 [33] was used to determine the compressive strength of the specimens (cube or sulfur-capped cylinder) [34]. The flexural properties of prisms measuring 160 × 40 × 40 mm were determined using a three-point bending test on an Instron (30-kN, Model 3367) machine [35]. The displacement rate used in this machine was 0.2 mm/min. The test setups for the mechanical properties are shown in Figure 14.

## 3. Results and Discussion

### 3.1. Effect of Aggregate Water Content

**Figure 15:**
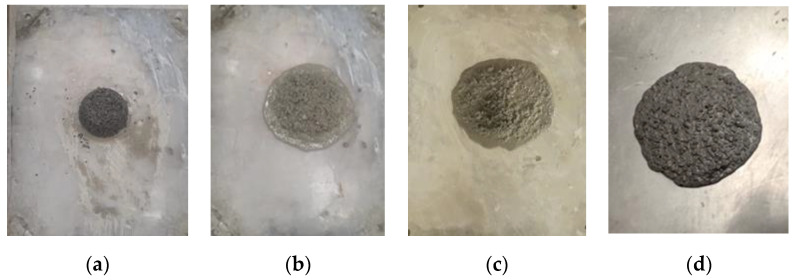
Effect of absorption water content on scoria aggregate performance (**a**) 100% water absorption, (**b**) 65% water absorption, (**c**) 25% water absorption and (**d**) 0% water absorption.

The effect of the degree of absorptivity of lightweight aggregates to mixing water, as indicated by an increase in strength with increasing lightweight aggregate content [36], was investigated indirectly. In their investigation, they explained that the improvement in strength as the result of water absorption by lightweight aggregates. The effect of the water content of aggregates is neglected. However, it is shown in the current investigation that the addition of absorption water to lightweight aggregates leads to a significant disturbance in the workability of SR mixes, as demonstrated in Figure 15. This phenomenon was ascribed to a reduction in surface tension near the aggregate as a result of the presence of excess water. It is well-reported that the plastic viscosity of the paste phase in concrete is determined by the surface tension of the water film [37]. The optical microscopic examination of the SR coarse aggregate confirms an increased capacity for cementitious matrix absorption with an adverse effect on workability. For this reason, a special mixing sequence of adding lightweight aggregates to already-prepared cement paste to increase viscosity was initiated and investigated.

### 3.2. Unit Weight Approach

**Figure 16:**
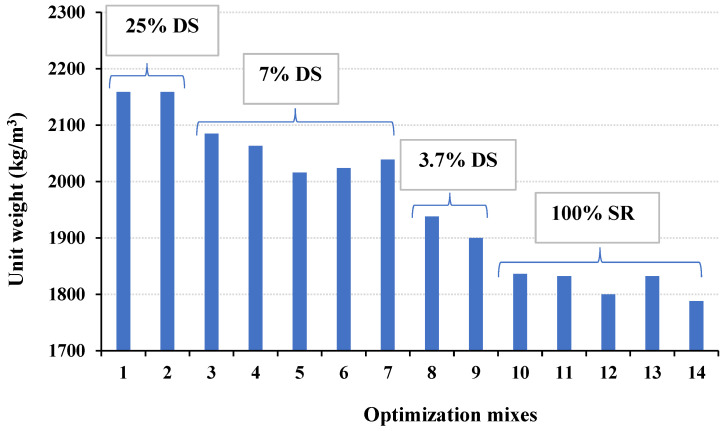
Effect of DS on the unit weight of the fresh concrete as the main pass-or-fail criterion.

Different trial mixes containing different contents of DS varied from 3.7 to 25% of the total volume of aggregates, compared to the mixes with 100% SR, and were prepared and tested for unit weight as the main criterion to test the fresh and hardened mix properties. All mixes with DS have surpassed a unit weight of 1900 kg/m^3^, as demonstrated in Figure 16. Therefore, the presence of DS was excluded from further investigation. The presence of DS appears to have increased mix compactness due to the spherical nature of its grains, as shown in Figure 9a [38]. The mixes with 100% fine and coarse SR aggregates were optimized for the lowest unit weight as well as fresh and hardened properties.

### 3.3. Effect Coarse-to-Total Aggregate Ratio on Flowability

**Figure 17:**
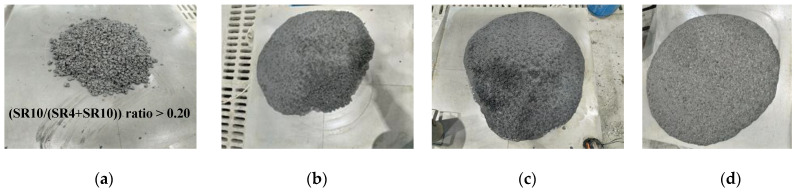
The effect of (SR10/(SR4 + SR10)) ratio on the flowability of SCC mixes with (**a**) 0.20 and (**b**) 0.16 and (**c**) 0.14 and (**d**) 0.125.

In this part of the study, the effect of different coarse aggregate-to-total aggregate ratios (R = SR10/(SR4 + SR10)) of 0.125, 0.14, 0.16, and 0.2 under constant superplasticizer dosage on flowability was evaluated, as shown in Figure 17. From the figure, it is concluded that the ratio of coarse aggregate content to that of total aggregate should not exceed 13%, as it dramatically affects flowability. This observation was attributed to the surface harshness of coarse aggregates, which become in need of a higher cement paste content to cover for better flowability. Under constant cement paste content, the higher ratio of the coarse aggregate of 0.2 showed no flowability at all, as depicted in Figure 17a. By reducing this ratio to 0.16, the mix became flowable but of low quality, as depicted in Figure 17b. Notably, the critical ratio is below 0.14, as confirmed in Figure 17c, while the optimum is at a ratio of 0.125, as demonstrated in Figure 15d.

### 3.4. Adjusting Superplasticizer Dosage

**Figure 18:**
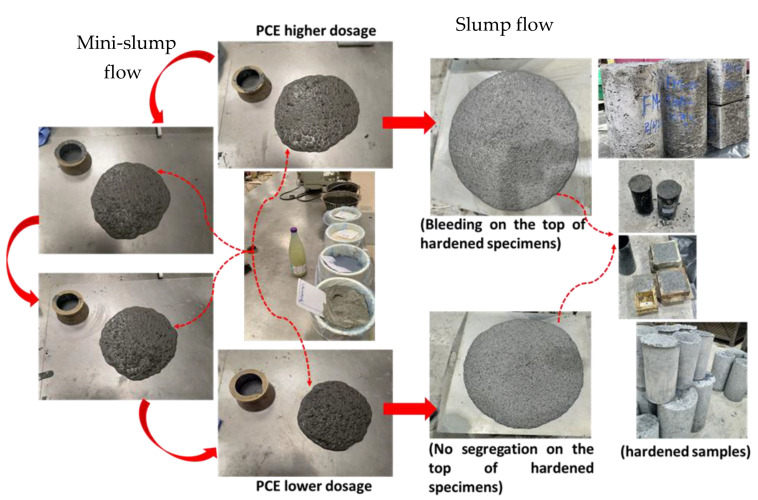
Cyclic optimization process from mini-slump to regular SCC slump flow.

Different dosages of superplasticizer (0.51, 0.61, 0.71, and 0.74% as a dry extract of total binder) were used to assess their effect on the regular flowability and segregation of LWSCC mixes. This test was conducted on a mini-scale first and verified on a normal scale, as shown in Figure 18. The results confirmed that the optimum superplasticizer dosage was about 0.51%. This dosage was further adjusted to obtain the optimal value at a dosage of 0.52%.

### 3.5. Final Mix

Based on the optimized parameters from previous sections of this study, the following final mix composition could be obtained, as shown in Table 4. In this final mix, a coarse-to-total aggregate ratio of about 0.13 was used. The water-to-binder ratio was 0.31, and the superplasticizer dosage was 0.52 (dry extract per binder content). The cement content was about 55%, while FA and SF contents of 18.5% and 7%, along with 19.6% LSD, were the other powders constituting the overall cementitious powder. It is worth mentioning that fine materials in scoria aggregates represent about 7% of the whole mix weight, which is composed mainly of fine scoria rocks with pozzolanic properties and the same mineralogical composition, as shown in Figure 1.

#### 3.5.1. Paste Composing LWSCC Mix

**Figure 19:**
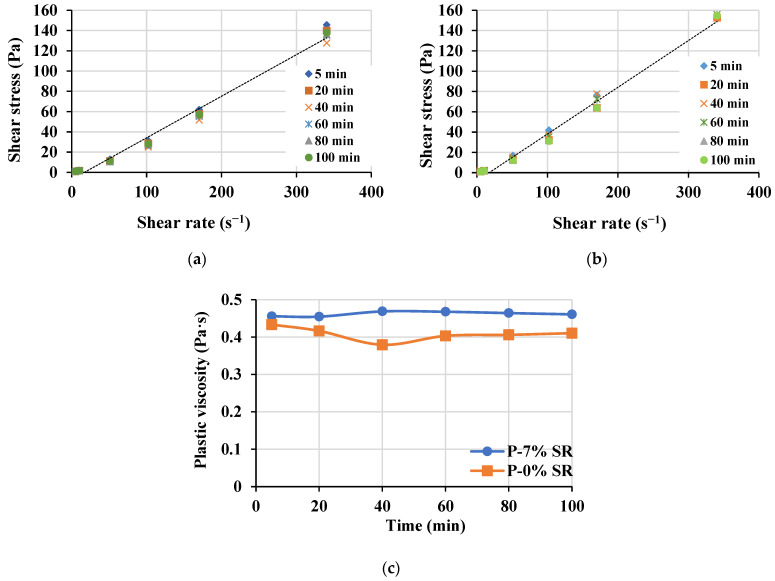
Rheological properties of paste mixes (**a**) without the fine materials (P-0% SR), (**b**) with 7% fine materials sieved from scoria rocks passing sieve 75 µm (P-7% SR) and (**c**) the evolution of the corresponding plastic viscosity over time.

The rheology of the paste phase with and without the fine powder from SR4 and SR10 below 75 µm was determined as shown in Figure 19. The relationship between shear rate and shear stress of the paste mixes without and with fine powder equivalent to that contained in scoria rocks is shown in Figure 19a,b, respectively. The presence of fine powder from SR aggregates has slightly increased the plastic viscosity. The plastic viscosity, however, has been shown to stabilize over time until 100 min, regardless of the presence or absence of fine powder from SR aggregates, as an indication of the workability retention, as demonstrated in Figure 19c.

**Figure 20:**
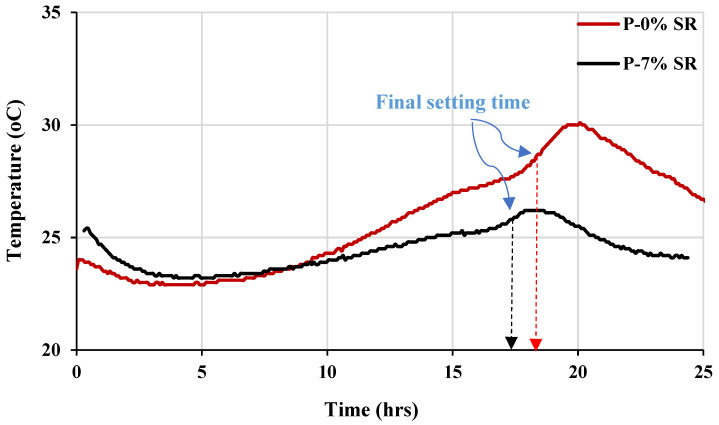
Temperature profiles of paste mixes without the fine materials (P-0% SR) and 7% fine materials sieved from scoria rocks passing sieve 75 µm (P-7% SR).

The temperature profiles of the two paste mixes without and with fine powder from SR aggregates are shown in Figure 20. The presence of SR powder has led to a significant reduction in the temperature peak, which has also shifted to a lower time, as demonstrated in the figure. This reducing effect has been attributed to lowered cement content and the effect of SR powder on the hydration mechanism, as confirmed in a previous study [21,22,23,24].

#### 3.5.2. Final Concrete Mix

##### Fresh Properties

**Figure 21:**
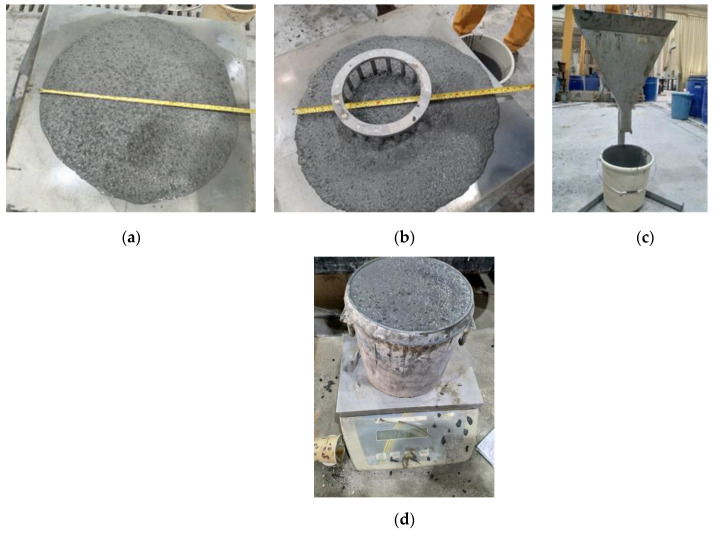
Typical measurement tests for (**a**) the slump flow and T50, (**b**) J-ring flow, and (**c**) V-funnel flow time and (**d**) unit weight determination.

The typical fresh properties of the final mix with the composition shown in Table 5 are presented in Table 5 and Figure 21. The test values are presented in Table 5, as per the EFNARC accepted range [39]. The slump flow values varied between 790 and 800 mm, while the corresponding slump flow time (T50) varied between 3.78 and 5.67 s. The J-ring flow values in the presence of steel bars vary between 750 and 780 mm. The unit weight measurement of different samples in a container with a capacity of 7L varied from 1790 to 1842 kg/m^3^. Owing to the reported values of the equilibrium density, which fall within the ACI 318 range of 1441.7 to 2162.5 kg/m^3^, the current concrete mix has been classified. Various density values as per the measuring conditions of the test mixes could be calculated. The range of fresh densities varied between 1790 and 1842 kg/m^3^. The saturated density varied between 1905 and 1928 kg/m^3^. Meanwhile, the air-dry density at a relative humidity of 20% and the saturated density at a relative humidity of 50% were in the ranges of 1757–1792, and 1770–1800 kg/m^3^, respectively. The oven-dry density obtained the lowest values in the range of 1707–1750 kg/m^3^, as lightweight concrete [30]. Various density values as per the measuring conditions of the test mixes could be calculated. The range of fresh densities varied between 1790 and 1842 kg/m^3^. The saturated density varied between 1905 and 1928 kg/m^3^. Meanwhile, the air-dry density at a relative humidity of 20% and the saturated density at a relative humidity of 50% were in the ranges of 1757–1792, and 1770–1800 kg/m^3^, respectively. The oven-dry density obtained the lowest values in the range of 1707–1750 kg/m^3^. It is reported that the accepted variation in the fresh density to the target value would be in the typical range of ±65 kg/m^3^ [40,41].

**Figure 22:**
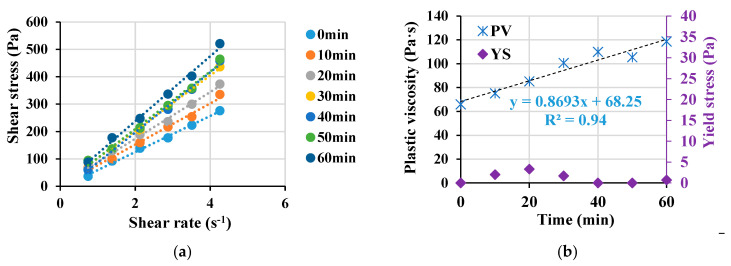
Rheological properties of LWSCC concrete mix (**a**) shear stress versus shear rate and (**b**) plastic viscosity(PV) and yield stress (YS) as a function of time.

**Figure 23:**
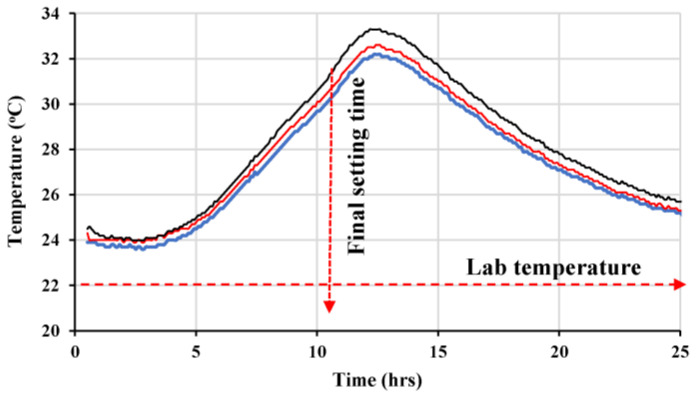
Temperature profiles of the final LWSCC mix (profiles of 3 specimens).

**Figure 24:**
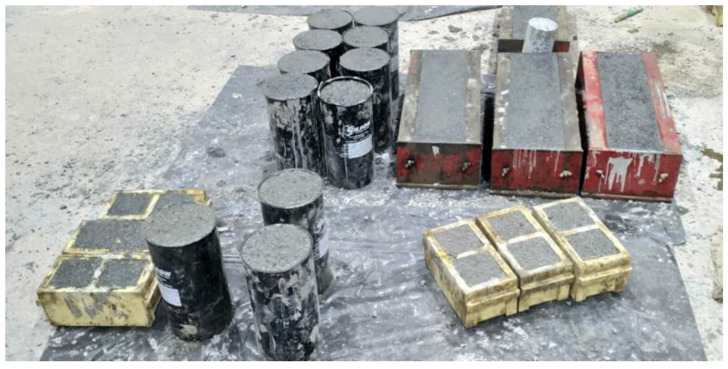
Typical samples cast for different mechanical properties.

The rheological properties of the mix, represented by the shear rate versus shear stress, are shown in Figure 22a, while the evolution of both the plastic viscosity and yield stress is shown in Figure 22b. The plastic’s viscosity increases significantly over time until 60 min. However, the yield stress is maintained at lower values with slight variation due to the high content of cementitious matrix and the low coarse-to-total aggregates ratio of about 0.13. The temperature profile of three concrete cubes (100 × 100 × 100 mm) was monitored for 24 h, and the results are shown in Figure 23. The temperature peak was reached at 12.6 h, while the estimated setting time was at about 10 h, as depicted in Figure 23. This refers to the finding that the optimal superplasticizer dosage did not negatively affect the setting time of the final concrete mix. The typical cast specimens are presented in Figure 24.

##### Hardened Properties

- Oven-dry unit weight

**Figure 25:**
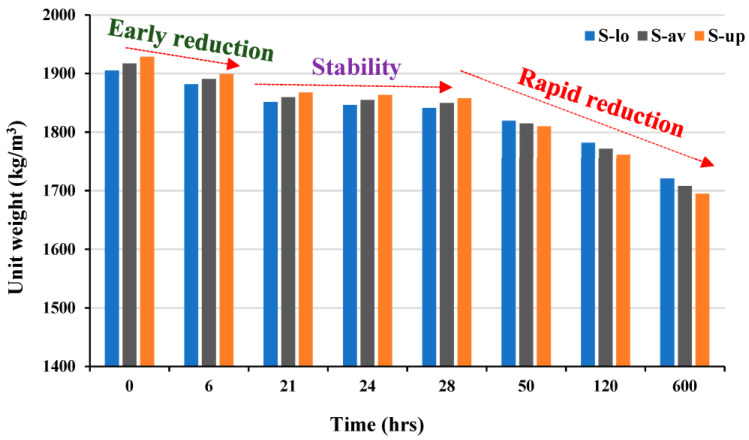
Variation of oven-dry unit weight over time of 2 specimens (low value = S-lo, upper value = S-up and the average value S-av).

It is shown that scoria rock grains are formed of vesicles bridged together in such a way that light reflection inside these vesicles can be easily monitored, as presented in Figure 4e. The presence of these vesicles imparts lightweight properties to scoria rocks. The water absorptivity of scoria grains is high accordingly, which can be a source of misconception in calculating the water-to-cement or even water-to-binder ratio correctly. The idea of this work was to make the mixing water unavailable for these vesicles. Accordingly, these cavities will, on the one hand, remain empty to keep the density as low as possible, while on the other hand, the mixing water will be maintained for the binder only, and accordingly, the water-to-cement ratio remains accurate. This approach was achieved by developing a special sequence of mixing with adjusted viscosity to minimize the absorptivity of scoria grains. This approach is shown to be unique through the literature review’s verification. Water tightness is one of the strongest tools to confirm this approach. For this purpose, the test in this part of the study was conducted to affirm this idea. The oven-dry unit weights of the specimens that had been water-cured were measured over time at a temperature of 100 ± 5 °C, as depicted in Figure 25. This protocol of testing confirmed that the lowest oven-dry unit weight at 24 h is 1846 kg/m^3^, while that at 600 h has reached 1694 kg/m^3^. If the water is entrapped inside the vesicles, the time needed should be much less than this reported long period of time. The unit weight value at 24 h is higher than that reported due to the prolonged curing time in water, which provided enough hydration time to make it difficult for the free water to escape during the early hours of oven treatment. However, the early reduction was attributed to the large pores. Once the finer pores are emptied, the stability of the unit weight shows a substantial reduction until 600 h, followed by stability for a longer time.

b Compressive and flexural properties.

**Figure 26:**
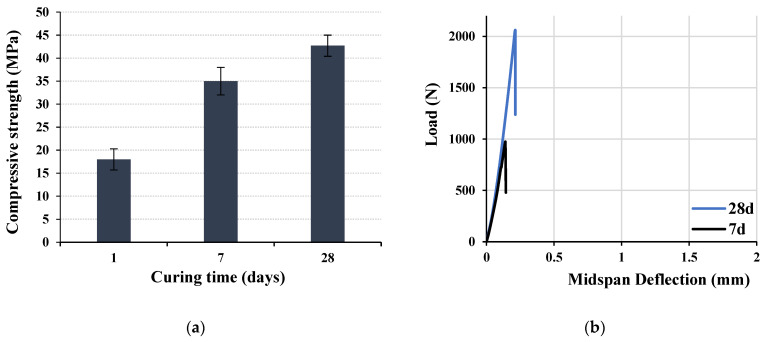
Development of the optimized mix specimens’ (**a**) compressive and (**b**) flexural strength.

The development of the compressive and flexural strengths of the optimized mix specimens with an R ratio of about 0.12 is shown in Figure 26a,b, respectively. The average maximum compressive strength at 28 days is about 42.7 MPa, while the corresponding flexural load of over 2000 N is attained. These values satisfied the pre-designed requirements in agreement with previous research [12,13,14,26,27]. It is accordingly concluded that structural LWSCC could be obtained using 100% scoria aggregates. An extension of this work is proposed for structural applications in different beams with variable dimensions. A total of three concrete beams measuring 150 × 150 × 600 mm were prepared, as shown in Figure 24, and tested according to ASTM C78 to calculate the modulus of rupture (MOR) [42]. The average value of the MOR was about 6.2 MPa. These encouraging results confidently recommend proceeding with investigating and testing the developed LWSCC for structural applications.

## 4. Conclusions

From the results of this study, the following can be concluded:

1. The effect of the degree of absorptivity of lightweight aggregates on mixing water becomes critical in the case of using 100% scoria aggregates. For this reason, a special mixing sequence of adding lightweight aggregates to already-prepared cement paste to increase viscosity and prevent water absorption from the mix was initiated and investigated.
2. The presence of DS increases the compactness of the mix due to the spherical nature of its grains and consequently increases the unit weight to pass over 1900 kg/m^3^.
3. The effect of different coarse aggregate-to-total aggregate ratios (R = SR10/(SR4 + SR10)) was shown to be critical, with an optimum ratio of 0.125 that delivered a successful LWSCC mix.
4. The final LWSCC mix could be categorized as low-cement concrete with acceptable fresh, rheological, and hardened properties both in the paste and concrete phases.
5. The modulus of rupture of 6.2 MPa encourages proceeding with investigating and testing the developed LWSCC for structural applications.
6. Finally, structural LWSCC can be obtained using 100% scoria aggregates.

## 5. Recommendations

The insertion of different types and contents of manufactured and recycled fibers is recommended for further investigation in these types of LWSCC mixes for advanced structural application.

## Figures and Tables

**Table 1:** Chemical analysis of different SR powders from different regions.

Oxides (%)	PC	SF	FA	LSD	SR
SiO_2_	20.41	91.3	53.2	1.80	43.31
Al_2_O_3_	5.32	0.35	27.3	0.45	15.41
Fe_2_O_3_	4.1	0.05	4.03	0.66	12.48
CaO	64.14	0.15	0.90	54.41	9.26
MgO	0.71	0.75	0.60	0.45	10.1
SO_3_	2.44	0.86	0.20	0.46	0.06
P_2_O_5_	0.04	0.06	0.40	0.01	0.4
TiO_2_	0.3	-	1.52	0.04	2.19
MnO	0.07	0.03	0.03	-	0.19
Na_2_O	0.10	0.40	0.22	0.06	2.95
K_2_O	0.17	0.80	1.22	0.03	0.75
Cl	0.01	0.10	0.05	0.03	0.03
L.O.I	2.18	5.1	10.02	41.6	0.95
D50	10	0.23	10	19	13
Sp. Gr.	3.15	2.2	2.6	2.7	2.8

**Table 2:** Location of collection site of SR samples.

Sample	GPS Coordinates	
	Latitude (N)	Longitude (E)
Scoria rock	23° 11.9103	39° 56.9014

**Table 3:** Mix design of the optimized LWSCC paste.

PC	FA	SF	LSD	W
442	149	57	158	253

**Table 4:** Mix design of the optimized LWSCC based on 100% SR aggregates.

PC	FA	SF	LSD	SR4	SR10	W
442	149	57	158	644	94	253

**Table 5:** Fresh properties of the final LWSCC mix.

	Tested Values	EFNARC Accepted Range; 2002 [39]
Slump flow (mm)	790–800	650–800
T50 (sec)	3.78–5.67	2–5 * 3–7 **
J-ring height (mm)	8–11	6–12
J-ring flow (mm)	750–780	-
V-funnel flow time (sec)	9.17	6–12

* Housing applications. ** Civil Engineering applications.

## Data Availability

Data available upon request.
